# Spatial co-occurrence of firearm homicides and opioid overdose deaths in Chicago by level of COVID-19 mortality, 2017–2021

**DOI:** 10.1186/s40621-024-00515-3

**Published:** 2024-07-31

**Authors:** Suzanne G. McLone, John R. Pamplin II, Jaii D. Pappu, Jaimie L. Gradus, Jonathan S. Jay

**Affiliations:** 1 Department of Epidemiology, Boston University School of Public Health, 715 Albany St., Talbot Building, 3E, Boston, MA 02118 USA; 2 Department of Epidemiology, Columbia University Mailman School of Public Health, New York, NY USA; 3 Center for Opioid Epidemiology and Policy, New York University Grossman School of Medicine, New York, NY USA; 4 Boston University, Boston, MA USA; 5 Department of Community Health Sciences, Boston University School of Public Health, Boston, MA USA

**Keywords:** Firearm, Homicide, Opioid overdose, COVID-19, Health disparity

## Abstract

**Background:**

Firearm homicide and opioid overdoses were already leading causes of death in the U.S. before both problems surged during the COVID-19 pandemic. Firearm violence, overdoses, and COVID-19 have all disproportionately harmed communities that are socially and economically marginalized, but the co-occurrence of these problems in the same communities has received little attention. To describe the co-occurrence of firearm homicides and opioid overdose deaths with COVID-19 mortality we used 2017–2021 medical examiner’s data from Chicago, IL. Deaths were assigned to zip codes based on decedents’ residence. We stratified zip codes into quartiles by COVID-19 mortality rate, then compared firearm homicide and fatal opioid overdose rates by COVID-19 quartile.

**Findings:**

Throughout the study period, firearm homicide and opioid overdose rates were highest in the highest COVID-19 mortality quartile and lowest in the lowest COVID-19 mortality quartile. Increases in firearm homicide and opioid overdose were observed across all COVID-19 mortality quartiles.

**Conclusions:**

High co-occurrence of these deaths at the community level call for addressing the systemic forces which made them most vulnerable before the pandemic. Such strategies should consider the environments where people reside, not only where fatal injuries occur.

## Introduction

Firearm-related homicides and opioid overdose deaths represent concurrent public health crises in the United States, each disproportionately impacting socially, racially, and economically marginalized communities (Barrett et al. 2022; Beard et al. 2017; Altekruse et al. 2020; Khatri et al. 2021). A previous study by Magee et al. has indicated that these fatalities tend to co-occur in the same neighborhoods (Magee et al. 2022). During the COVID-19 pandemic, both firearm homicides and opioid overdose deaths sharply increased, reaching record levels in many U.S. cities (Collaborative and [Internet]. 2023; Haley and Saitz 2020). Since firearm purchases (Schleimer et al. 2021) and the likelihood of using drugs in isolation (Nguyen and Buxton 2021) increased during the pandemic, it was not surprising that these deaths rose at the same time COVID-19 losses mounted. However, it remains unknown whether these trends co-occurred in the same communities, *i.e.,* whether firearm homicide and opioid overdose death rates varied by levels of COVID-19 mortality. This information is important because communities where all three types of losses occurred are in need of more resources to address prevention and aftereffect services in the face of the COVID-19 pandemic. In order to examine the co-occurring trends geographically, we describe the incidence of firearm homicides and opioid overdose fatalities in Chicago, Illinois before and during the COVID-19 pandemic according to neighborhood-level COVID-19 mortality.

## Methods

We obtained data from the Cook County Medical Examiner Open Source Data Archive (Cook County Medical Examiner | Cook County Government | Open Data 2023) on the cause and manner of deaths from 2017 to 2021. We incorporated pre-pandemic data to allow examination of how trends may have changed during the pandemic. In 2015–16, there was an unusual spike in firearm homicides in Chicago, (Cassell and Fowles 2018) so we used 2017 as the starting point for pre-pandemic trends. The study population included decedents who were residents of Chicago, Illinois and died in 2017–2021 for firearm homicides and accidental opioid overdose deaths, or who died in 2020–2021 for COVID-19 deaths.

The geographic unit of analysis was decedent residential zip code. We used residential zip code, rather than zip code where the injuries occurred, as a better proxy for the community where a loss was experienced and where decedents may have been exposed to relevant risk factors.

We aggregated the counts of COVID-19 deaths from 2020 and 2021 by decedent residential zip codes (n = 62). To generate crude rates per 10,000, we obtained 2017–2021 5-year population count estimates from the American Community Survey (United States Census Bureau 2023). These zip code-level rates of COVID-19 mortality were categorized into quartiles (hereafter “COVID-19 quartiles”) which were selected to distribute the data evenly and to ensure that each group had sufficient data to generate stable rates.

To capture changes from pre-pandemic mortality levels, we calculated firearm homicide and opioid mortality rates using the same procedure as for COVID-19 but included data going back to 2017. These rates were stratified by COVID-19 quartile. Average annual mortality rates per 10,000 and 95% confidence intervals were calculated by COVID-19 quartile for both firearm homicides and opioid overdose deaths. Mortality rates were not age adjusted, because our primary focus was the overall burden of loss across communities. Also, differences in age distribution across neighborhoods might mask spatial co-occurrence between firearm homicides and opioid overdose deaths with COVID-19 deaths, given that older age is a significant risk factor for COVID-19 mortality but not for the other outcomes. Conservatively, we calculated confidence intervals as if deaths were obtained from a sample of the population, though medical examiners are meant to document all deaths from firearm injuries and overdoses.

## Results

In the final analytic dataset, there were 5,867 COVID-19 deaths, 2,573 firearm homicides, and 3,996 opioid overdose deaths. For the combined years of 2017–2021, the mean firearm homicide rate was 1.72 (*SD* = 2.20) per 10,000 and the mean opioid overdose rate was 3.05 (*SD* = 3.21) per 10,000.

**Fig. 1:**
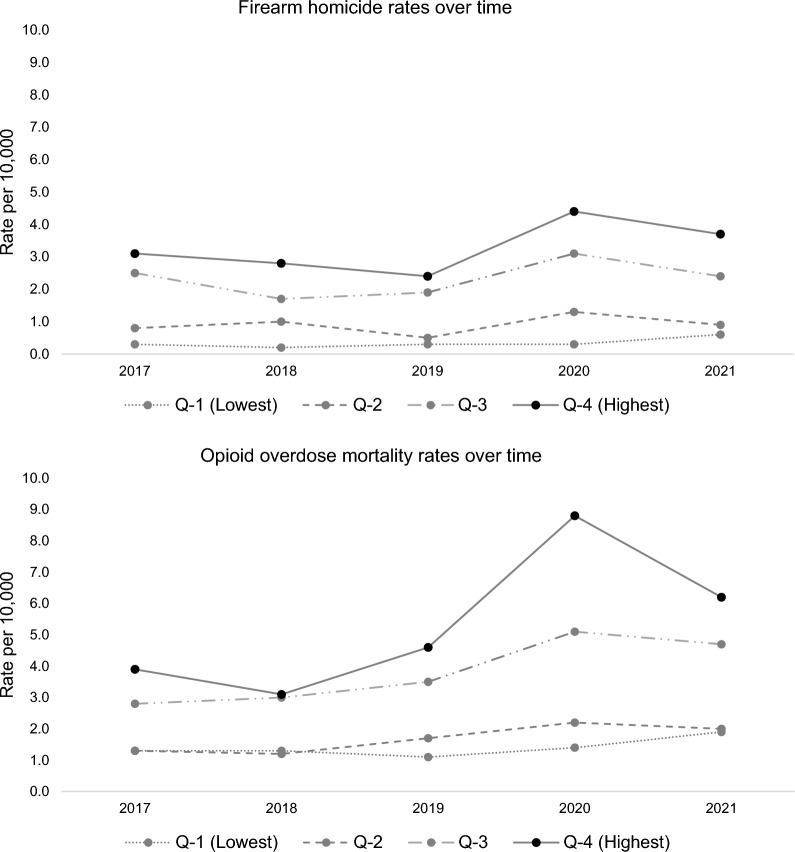
**Panel 1** Firearm homicide and **Panel 2** opioid overdose mortality rates in Chicago by year and COVID-mortality quartile, 2017–2021. Notes: Q = COVID quartile; all deaths coded by residential zip code of decedent

The mean COVID mortality rate was 9.23 (*SD* = 5.66), with means ranging from 1.71 (*SD* = 1.44) in the lowest quartile to 16.26 (*SD* = 2.45) in the highest quartile. Firearm homicide and opioid overdose mortality rates were greater in the quartiles representing higher COVID-19 mortality, both before and during COVID-19 (Fig. 1). Overall, COVID-19 mortality was aligned with firearm homicide and opioid overdose. Both firearm homicide and opioid overdose rates in the highest-COVID quartile were greater than in the lowest-COVID quartile throughout the study period.

In 2017, the firearm homicide rate per 10,000 in the highest COVID-19 quartile was 3.1 (95% CI: 1.6, 4.5), and in the lowest COVID-19 quartile was 0.3 (95% CI: 0.0, 0.6). In 2020, the firearm homicide rate per 10,000 in the highest COVID-19 quartile was 4.4 (95% CI: 2.3, 6.4), and in the lowest COVID-19 quartile was 0.3 (95% CI: 0.0, 0.7). In 2017, the opioid overdose mortality rate per 10,000 in the highest COVID-19 quartile was 3.9 (95% CI: 1.9, 5.9), and in the lowest COVID-19 quartile was 1.3 (95% CI: 0.2, 2.4). In 2020, the opioid overdose mortality rate per 10,000 in the highest COVID-19 quartile was 8.8 (95% CI: 3.6, 14.0), and in the lowest COVID-19 quartile was 1.4 (95% CI: 0.5, 2.3). In other words, mortality rates for firearm homicide and opioid overdose were nearly unchanged in the lowest COVID-19 quartile, while the highest COVID-19 quartile experienced substantial increases in both outcomes.

## Discussion

In Chicago, the geographical areas most impacted by COVID-19 deaths were also most impacted by firearm homicides and opioid overdose deaths. Communities with the highest COVID-19 mortality also had the highest rates of firearm homicide and opioid overdose pre-pandemic and the most pronounced increases during the pandemic.

Our results build upon scant previous work looking at the spatial co-occurrence of firearm homicides and opioid overdose deaths by adding another dimension: COVID-19. High rates of all three outcomes among residents of the same communities suggests the burden of loss was distributed unequally, even as research documents the widespread psychological harms of the pandemic period (Daly and Robinson 2021).

The finding that COVID-19 mortality was highest in communities with the highest pre-pandemic rates of firearm homicide and opioid overdose suggests that some of the same factors influenced vulnerability to these harms. Shared social determinants (*e.g.,* economic hardship, housing insecurity) and structural forces (*e.g.,* segregation, structural racism) likely contributed to the co-occurrence of risk for each outcome. These should be a topic of future research.

A strength of this study is that we use Medical Examiner Data, where the cause of death and manner of death are determined according to national standards (Inspection and Accreditation Checklist). Additionally, all three types of death were under the purview of the Cook County Medical Examiner during the study period, so it is unlikely that few, if any, of these deaths were not evaluated by the Cook County Medical Examiner.

This study has some limitations. Residence of decedent is determined by identification, *e.g.* driver’s license, and/or decedent’s next of kin. Therefore, it is possible the decedent was living at a location other than the one listed at the time of death. The catchment area of location of death is Cook County, Illinois, therefore it is possible that some Chicago residents died by firearm homicide outside of Cook County and are not included in this study.

These results suggest firearm homicides, opioid overdose deaths, and COVID-19 deaths are associated at the community level. Communities which were already experiencing the highest rates of firearm violence and opioid overdose deaths additionally experienced the highest rates of death during a new public health crisis, COVID-19. The findings of this study also emphasize the relevance of the decedent's residential location in examining the spatial distribution of firearm homicides and opioid overdose deaths, rather than solely focusing on the location of fatal injury.

At present, the structural factors and socioeconomic drivers that make these communities more likely to experience these fatalities, and have the least access to support services and care, are the same (Barrett et al. 2022; Beard et al. 2017; Altekruse et al. 2020; Khatri et al. 2021) This further emphasizes the importance of focused interventions within these communities.

Preventive strategies to support populations made vulnerable by social inequities should include both community-based and place-based interventions, with consideration for individuals’ residential context in examining the spatial distribution of firearm homicides and opioid overdose deaths. For example, income support offered to residents in socioeconomically marginalized communities have shown promising results in reducing interpersonal firearm violence (Rowhani-Rahbar et al. 2022). Local health departments frequently face financial deficits and a scarcity of evidence-based medications for opioid use disorder, indicating that sufficient funding for and procurement of these medications represents an obvious policy remedy (Feuerstein-Simon et al. 2020). Furthermore, policies aimed at reducing firearm homicides and opioid overdoses need to focus on improving access to mental health and substance use treatment in communities already burdened by increased exposure to environmental risk factors, systemic racism, and inequitable access to healthcare systems.

## Data Availability

Data used for this study are publicly available for download at Cook County Government Open Data, Medical Examiner Case Archive: https://datacatalog.cookcountyil.gov/Public-Safety/Medical-Examiner-Case-Archive/cjeq-bs86/about_data.
